# Evaluation of acute flaccid paralysis surveillance system in the River Nile State - Northern Sudan, 2021

**DOI:** 10.1186/s12889-023-15019-w

**Published:** 2023-01-18

**Authors:** Alhaj Saad Mohamed Ahmed Ali, Haghamad Allzain, Omran M. Ahmed, Elsadig Mahgoub, Mazin Babekir Musa Bashir, Babbiker Mohammed Tahir Gorish

**Affiliations:** 1 Department of Public Health, Faculty of Public Health, Shendi University, Shendi, Sudan; 2 Department of Biochemistry, Faculty of Medicine, Shendi University, Shendi, Sudan; 3 AFP Surveillance System, Federal Ministry of Health, Khartoum, Sudan; 4 Department of Microbiology, Faculty of Medical Laboratory Science, Shendi University, Shendi, Sudan; 5 Department of Microbiology, Faculty of Medical Laboratory Science, Omdurman Islamic University, Omdurman, Sudan

**Keywords:** Acute flaccid paralysis (AFP), Surveillance system, Polio, Sudan

## Abstract

**Background:**

One of the four main elements of the worldwide polio eradication strategy is acute flaccid paralysis surveillance (AFP). This system is based on (acute flaccid paralysis (AFP) cases reported and tested at World Health Organization (WHO) accredited laboratories. To measure and monitor performance, indicators were created. The current study aims to evaluate the system components, performance, and efficiency in River Nile State, Northern Sudan, and their compliance with World Health Organization (WHO) requirements for it to be adopted as a good system; its results can be used to certify whether a country is polio-free or not.

**Material and methods:**

A facility-based retrospective descriptive study was conducted in the River Nile State, Northern Sudan, from Jan 2017 to Dec 2020. This study included all reporting sites/units, workers who reported acute flaccid paralysis (AFP) cases, and officers at the locality level. A total of 50 health institutions were visited for surveillance, and interviews with 59 health workers who were part of the AFP surveillance system were undertaken. The data were collected from participants using a pre-tested questionnaire designed and constructed by the World Health Organization (WHO) framework, and the data were analyzed using the SPSS version (22).

**Results:**

The River Nile State’s AFP surveillance system was of high quality in terms of the infrastructure that had been put in place and the effectiveness of the system’s operations, as evidenced by the following statistics: from 2017 to 2020, the reported non-polio acute flaccid paralysis (AFP) cases were at a mean rate of 4.02 per 100,000 children under the age of 15; the majority of AFP reported cases were under 10 years; and males made up 73.3% of reported cases; The completeness of reports and surveillance documents exceeded 80%, and active surveillance was applied in 80% of reporting sites.

**Conclusion:**

Despite the fact that the surveillance system is capable of detecting cases, Sudan continues to report cases of imported polio from other countries, highlighting the need to strengthen surveillance systems and eradication efforts in these countries.

## Introduction

Surveillance is a critical component of the polio eradication effort. Without surveillance It would be hard to determine where and how wild and circulating vaccine derived poliovirus is still spreading or to confirm that the virus has been eradicated. Surveillance finds new cases and detects wild poliovirus importation [1]. Poliomyelitis surveillance provides data for programmatic intervention. It is used to measure the efficacy of immunization program and to guide attempts to eradicate the disease and the virus. The detection, reporting, clinical evaluation, and virological testing of stool specimens from all individuals with acute flaccid paralysis form the basis of surveillance acute flaccid paralysis (AFP). Public health workers, doctors, epidemiologists, and virologists must work together to ensure successful surveillance.

Acute flaccid paralysis (AFP) surveillance provides evidence for certification of eradication. To document the absence of wild viral isolates from acute flaccid paralysis (AFP) patients, healthy children, and the environment, validated sensitivity surveillance technologies are necessary. A global network of high-quality poliovirus laboratories capable of identifying wild virus when and where it appears is critical to poliovirus eradication and certification. Following the worldwide resolution of 1988, establishment of the network began in 1986 in the Americas, the first region to declare its goal to eradicate poliomyelitis and has proceeded in other World Health Organization (WHO) regions [2]. Polio eradication requires good surveillance systems. They enable the detection of any remaining viruses and serve as a central point for eradication verification. This section lays out the procedures that will be required to continue to create and maintain a robust polio monitoring system to eradicate the disease [3].

Acute flaccid paralysis (AFP) is a clinical illness characterized by the sudden development of limb weakness, described as flaccid (reduced tone) in a child under the age of 15 [4]. Because acute flaccid paralysis (AFP) resembles the clinical appearance of poliomyelitis, it has been adopted as a crucial tool for tracking the progress of the global polio eradication effort [5]. To guide programmatic action, any disease eradication campaign relies on highly sensitive surveillance. This is especially essential for polio eradication because only one out of every 200 poliovirus infections result in clinically evident paralysis [6]. Surveillance must discover and examine as many instances of paralytic poliomyelitis as feasible to locate and eliminate remaining pockets of wild poliovirus transmission. To accomplish eradication, high levels of polio vaccine coverage must be maintained over the world, which is reliant on a sufficient vaccine supply [6].

A strong acute flaccid paralysis (AFP) surveillance system acts as a sensitive tool for detecting possible poliomyelitis cases, alerting health managers and physicians to implement relevant actions promptly to prevent poliovirus transmission. Effective acute flaccid paralysis (AFP) surveillance is also necessary for confirming the absence of wild poliovirus circulation in areas where instances of poliomyelitis are no longer reported [7]. The World Health Organization (WHO) has taken up acute flaccid paralysis (AFP) surveillance to track progress toward poliomyelitis elimination. Acute flaccid paralysis (AFP) surveillance aids in the identification of poliovirus transmission hotspots. As a result, acute flaccid paralysis (AFP) surveillance data will be used to guide targeted immunization efforts in areas where wild poliovirus is still circulating. Surveillance data is also widely considered the most trustworthy tool to track how well routine and additional oral polio vaccine (OPV) immunization has reduced poliovirus transmission. In the end, surveillance data will serve as the foundation for polio eradication certification [7].

Insecurity, forced displacement, frequent nomadic population movement, and inaccessibility in some regions pose problems to the surveillance system in Sudan, making it difficult for health professionals to consistently access all children with vaccines to establish immunity. Special initiatives have been established at the state, locality, and administrative unit levels to address specific surveillance issues associated with reaching high-risk groups. Active searches for acute flaccid paralysis (AFP) cases and sample collection by community-based surveillance officers in areas with limited access, mapping of displaced populations’ movements, and maintaining regular communication with nomadic community focal points who report acute flaccid paralysis (AFP) cases via mobile phone are just a few examples. Vaccination stations in refugee camps have allowed for the screening of children with acute flaccid paralysis (AFP), and collaboration and sensitization of non-governmental organization (NGOs) employees have helped to enhance acute flaccid paralysis (AFP) case reporting [8].

The acute flaccid paralysis (AFP) surveillance system is based on reporting and testing instances of acute flaccid paralysis in World Health Organization-accredited laboratories, where performance indicators have been developed. In Sudan the national laboratory and National Expert Committee play a major role in the quality of surveillance, which in turn contributes to the polio eradication. The national laboratory examines all samples sent to it, and sends the results of the tests it conducted, as well as the results of the tests conducted in the reference laboratories accredited by the World Health Organization to the National Administration for Expanded Immunization, which in turn informs the states of these results, which must send these results to the localities so that they can reach this information is for the person who initially identified the case (feedback), while The role of the National Expert Committee is to consider cases that do not have sufficient samples and have residual paralysis after 60 days of paralysis, or for which no samples were collected or any other cases that could not be classified by the laboratory. In this case, samples are collected from 5 contacts, a detailed investigation, a medical report, a nervous system examination, and any other documents or tests that help classify the case are completed. The committee reviews 5–10% of the cases that were excluded by the laboratory to confirm their classification [9].

In study area There is no study has been conducted to evaluate the acute flaccid paralysis (AFP) surveillance system in the past, necessitating the need to study the components of the system and evaluate its performance, efficiency, and conformity with the World Health Organization’s approved standards for it to be adopted as a good system, the results of which can be accredited to certify whether Sudan country is polio-free or not. This approach is the only way to demonstrate that Sudan is polio-free. Continuous surveillance of acute flaccid paralysis in the state; It is a task that requires skill and constant funding to achieve the goal of polio-free Sudan. Our study aimed to assess the structural components of acute flaccid paralysis (AFP) surveillance system, to assess the performance of core activities for acute flaccid paralysis (AFP) surveillance system and to identify the support elements of acute flaccid paralysis (AFP) surveillance system.

## Material and methods

### Study design

Between Jan 2017 to Dec 2020, a retrospective descriptive facility-based study was done to evaluate the acute flaccid paralysis surveillance system in the River Nile State of Northern Sudan. The state of River Nile is located north of Khartoum. It covers an area of 124 thousand square kilometers and is located between latitudes 16–22 North and longitude 30–32 East. It has a population of approximately (1,212,000). The state has approximately 1962 schools, three universities, and a handful of research organizations. There are approximately 34 hospitals and 156 health centers in the state.

### Study population

All reporting sites/units, “*N* = 50 sites” from all levels and workers who are reporting AFP cases, “*N* = 50 focal persons,“ locales officers, “*N* = 6 officers,“ and state level, “*N* = 3,“ were included in the research population. All health facilities conducting AFP monitoring in the state, focal persons working in health facilities, department officers in localities, and system coordinators at the state level were covered by the whole coverage of surveillance officers and workers in the AFP surveillance system.

### Data collection tools and analysis procedures

Multiple tools were used for this study including group discussion with system coordinators, a check list of observations, and analysis form for records and a questionnaire for officers and focal persons. The questionnaire was designed and developed according to the WHO framework for communicable diseases surveillance, it contains variables related to AFP surveillance as:

❖ Structure variables; including (organization, legislation, strategy, available offices, existing plans of action coordination, network, and lab facilities)
❖ Surveillance resources; including (doctors, public health officers, lab technicians, medical assistants, nurses and statisticians)
❖ Operational resources: including (financial, materials, and supplies) were Supplies include (register book, forms, case definition, guidelines, posters, transport and communication means)
❖ Cases detection and registration variables
❖ Diagnosis confirmation variables
❖ Reporting, information, dissemination, response and feedback variables
❖ Supervision variables
❖ Indicators of AFP surveillance system

The questionnaire was amended to suit the study method and the respondents. After designated the questionnaire in its first form, it was presented to specialists to assess the structure and face validity. Then it was distributed to some personnel as a small sample (*n* = 10) to measure the reliability and stability (Redistribute the questionnaire to the same group and give the same results).

#### Interview design

This tool is designed to interview AFP surveillance coordinators at the state level and contains the following:

❖ The health facilities selected for surveillance.
❖ Other sites than the health facilities covered by the surveillance system.
❖ The period between one visit and another is based on the type of centre (upper, middle, lower, and additional)
❖ Other sectors that participate in the surveillance system and their role in achieving the goals of the system and implementing its activities
❖ Challenges facing the system in the state in terms of material, human and legal resources, as well as society and its habits and culture

#### Check list design

This tool used to observe some elements that exist at all levels of the study population contains the following:

❖ AFP surveillance documents
❖ Concerned functions of AFP surveillance system

#### Data analysis

Data analyzed by using SPSS version 22; include multi of statistics needed in this study as:

❖ Frequencies tables: to show data include percentages and frequencies
❖ Cross tabulations; to know the relationship between two or more variables
❖ Chi- square crosstabulation test was used in some variables to know Significance between variables at *P*-value of 0.05 or less as significant value as:
  Correlation between Priority of reporting sites and complete of AFP documents
  Correlation between Case definition and specialty of the focal person
  Correlation between number of workshop and focal person’ skills
  Correlation between priority of reporting site and Supportive elements availability
❖ Alpha Cronbach’s test: It was performed as a pre-test of the study tool to find out the data collection tool stability.

#### AFP surveillance indicators

- A non-polio AFP rate of at least 1/100 000 children under 15 years of age
- Two adequate specimens collected from at least 80% of detected AFP cases
- All samples handled in a WHO-accredited laboratory
- Percentage of all expected monthly reports that were received: target ≥ 90%
- Percentage of AFP cases investigated within 48 h: target ≥ 80%
- Percentage of AFP cases with two adequate stool specimens collected 24–48 h apart and ≤ 14 days after onset: target ≥ 80%
- Percentage of specimens arriving at the laboratory in good condition: target ≥ 80%
- Percentage of specimens arriving at a WHO-accredited laboratory within three days of being sent: target ≥ 80%
- Percentage of specimens for which laboratory results were sent within 28 days of receipt of specimens: target ≥ 80%

## Results

The AFP surveillance system in the River Nile State was of high quality in terms of established infrastructure and performance of the system’s activities; the system’s quality is because it has an established infrastructure, as it is committed to the general strategy of AFP surveillance, as cases are mandatory reported in all reporting sites that are distributed by a high level of representativeness, which includes beside known health facilities (28 hospital (Table 1).

**Table 1:** Type and priority AFP reporting sites in River Nile State, Sudan, 2020, (*N* = 50)

Reporting cites	Priority				Total
	High	Middle	Low	Supplementary	
Hospitals	4	1	6	17	28 (56%)
Health centers	0	2	2	15	19 (38%)
Clinics	1	2	0	0	3 (6%)
**Total**	5 (10%)	5(10%)	8(16%)	32 (64%)	50 (100%)

All surveillance sites were divided into five categories: high priority (5 sites) include 4 hospitals and 1 clinic, medium priority (5 sites) include 1 hospital, 2 health centers and 2 clinic, low priority (8 sites) include 6 hospitals, 2 health centers, and additional surveillance sites (32 locations) include; 17 hospitals and 15 health centers (Table 1). Concerning system sensitivity, the surveillance system was found to be sensitive, with an average of 4 cases per 100,000 children under the age of 15 for the years 2017 to 2020, with a minimum of 4/100 000 in 2019, 2020 respectively and a maximum of 5/100 000 in both 2017 and 2018 (Table 2), and thus complying with the WHO standard of greater than or equal to two cases per 100,000 children under the age of 15. Males accounted for 73.3% of AFP-reported cases under the age of ten years (Table 2).

**Table 2:** Distribution of AFP reported cases according to sex/age and sensitivity

Sex /age	Mean of AFP cases/year/100,000 = 4				Total
	2017 5/100,000	2018 5/100,000	2019 4/100,000	2020 4/100,000	
Male (74%)					
Less than 5	6	7	7	8	28(33%)
5 to 10 years	8	9	11	4	32(37%)
11 to 15 years	0	2	0	2	4(5%)
Female (26%)					
Less than 5years	3	2	2	5	12(13%)
5 to 10 years	2	2	2	5	11(12%)
11 to 15 years	0	0	0	0	0
**Total**	19/380,000	22/440,000	22/575,000	24/600,000	86(100%)

The results of all case investigations are complete, and the reports (more than 80%) all meet WHO requirements (Table 3). The results of the activities showed that they were in line with the surveillance system’s general standards, as there was an easy-to-use AFP field guide for all workers in surveillance activities at all levels, which included guidelines for writing reports and the decision-making process, as well as the presence of focal persons in all reporting sites. The focus persons in the reporting sites have a variety of specialties, including 22% doctors, 14% nurses, 50% immunization officers, 2% nutritionists, and 12% statisticians (Table 4).

**Table 3:** The observed performance indicators and WHO performance targets

Performance indicators	Target	Indicator	Performance
Rate of Annual reported cases	≥ 2	Sensitive	4
Proportion of Investigated cases	≥ 80%	Completeness of investigation timeline	100
Proportion of AFP cases investigated < 48 h reported	≥ 80%	Timeliness	97.7%
Proportion of AFP cases followed up at 60 days	≥ 80%	Completeness of follow-up	95%
Proportion of AFP cases with two adequate stool specimens	≥ 80%	Timeliness	100%
Proportion of specimen results sent from national laboratory within 28 days of receipt of the specimen in the laboratory	≥ 80%	Timeliness of laboratory investigation	100%
Proportion of surveillance site providing routine report (including zero reports’) R2 on time	≥ 80%	Completeness of reporting	100%
Proportion of Active visits	≥ 80%	Completeness	84%

**Table 4:** Specialty and knowledge of focal persons about AFP case definition, (*N* = 50)

AFP Case definition	Specialty of Focal person					Total
	Doctor	Nurse	Immunization officer	Nutritionist	Statistician	
Good	11	7	24	1	6	49(98%)
Acceptable	0	0	1	0	0	1(2%)
**Total**	11(22%)	7(14%)	25(50%)	1(2%)	6(12%)	50(100%)

Our result demonstrated that 90% of focal persons are formally employed, 78% have deputies who perform their roles in the event of the absence of the focal person, and although 96% have received training, only 42% have computer skills, 40% have data entry skills, 38% have the skill of designing tables and graphics. 98% of the employees keep copies of previous reports, 84% indicated the existence of active visits, 60% confirmed that the visits were regular, and 52% of the signed visit form was noticed, (Table 5). Result on data management facilities demonstrated that Calculators were available for data analysis in all locality’s offices and 36% of health facilities, all localities had computers, and only 10% of health facilities had a computer, 24% of health facilities have a phone for surveillance activities, (Table 6). With regards to the relationship between focal person’s skills and training workshop our results demonstrated that when two training workshops were attended, computer skills were improved (Table 7).

**Table 5:** Core activities for AFP Surveillance system at health facilities level, (*N* = 50)

Core activities	Existed elements	
	No	Percent
Presence of AFP surveillance guideline	50	100%
An easy-to-use AFP surveillance guideline	50	100%
Uses the AFP surveillance guide in surveillance activities	50	100%
AFP surveillance guideline contains the report guideline	50	100%
AFP surveillance guideline contain decision making- guide	50	100%
Modern AFP surveillance guide-line guideline	50	100%
presence of focal person	50	100%
Employed focal person	45	90%
Presence of deputy for focal person	39	78%
Monitoring the cases in out clinic records	48	96%
Monitoring the cases in other records	37	74%
Presence of trained focal person	48	96%
Personnel have computer skills	21	42%
Personnel has data interring skills	20	40%
Personnel have design tables skills	19	38%
Personnel have good relationship	48	96%
Presence the copies of reports	49	98%
Presence of active surveillance visits	42	84%
Presence of systematic surveillance visits	30	60%
Presence of sign in the visit form	26	52%

**Table 6:** Supportive functions of AFP surveillance system at health facilities level, (*N* = 50)

Supportive functions	Observed supportive elements	
	No	Percent
Presence of separated AFP surveillance room	34	68.0%
Presence of AFP surveillance officers	43	86.0%
Presence of data entry officer	13	26.0%
Presence of AFP surveillance supervisor	47	94.0%
Presence of lab	40	80.0%
Presence of lab technician	40	80.0%
Presence of lab guide	40	82.0%
Presence of electricity	50	100.0%
Presence of transport car	3	6.0%
Presence of telephone	12	24.0%
Presence of calculator	18	36.0%
Presence of computer	5	10.0%
Presence of printer	0	0
Presence of an internet line	0	0
Presence of last version of AFP surveillance guide	48	96.0%

**Table 7:** Relationship between number of workshop and focal person’ skills

Focal persons skills	Number of workshop		*P* value
	one	two	
Focal personnel have computer skills			
yes	20	1	0.033
no	20	8	
Focal person has data interring skills			
yes	20	0	0.004
no	20	10	
Focal person with design tables skills			
yes	19	0	0.006
no	21	10	

## Discussion

The findings of this study revealed that the AFP surveillance system in the River Nile State was of high quality according to measures of completeness and sensitivity in terms of established infrastructure and system performance, the non-polio AFP cases in this study were observed as more than 2 cases per 100,000 children under 15 years, to be sensitive when compared to WHO performance indicators. The system’s exceptional sensitivity allows it to detect any polio case brought into the state [10]. The sensitivity of the system is because of an established infrastructure, as it is committed to the general strategy of AFP surveillance, as cases are mandatory reported in all reporting sites that are distributed by a high level of representativeness, which includes known health facilities (hospitals, health centers, and clinics). Other sites such as traditional healers and physiotherapy centers are one of community surveillance strategies [11]. However, hospitals on the other hand, are regarded as the most important sites for reporting by 56%, which is consistent with what was stated in the national guidelines for surveillance and investigation of acute flaccid paralysis, which stated that hospitals are the most important places chosen for investigation and surveillance of acute flaccid paralysis and that cases of acute flaccid paralysis frequently go to hospitals [9]. And also agreed with GPEI which mentioned the public health staffs make regular visits to hospitals and rehabilitation centers to search for AFP cases which may have been overlooked or misdiagnosed.

In areas with few formal health workers, some countries use community surveillance, where pharmacists, traditional healers or clerics may serve as a source of information on paralyzed children, [12]. All reporting sites report AFP cases through personnel trained in the reporting process, with varying specialties, but there are no statistically significant differences in their knowledge of the definition and detection of acute flaccid paralysis cases; as it was AFP investigation at a high level among all workers of various specialties, there are no statistically significant differences in their knowledge of the definition and detection of acute flaccid paralysis cases. It was better than other locations, such as the Bikita region of Zimbabwe, where the author stated that health professionals’ awareness of AFP was limited and that all employees questioned required training and supervision [13].

Despite the employee’s possession of information related to the system activities, there is weakness in some aspects, such as computer skills and the process of data entry and representation with a statistical relationship. Receiving more training improving skill of using the computer and entering data, may be due to workers’ lack of computer equipment in the reporting sites. This weakness and lack of skills may be explained by the decrease in the allocated budget and the absence of financial support for the program, which may have an impact on improving performance and encouraging employees to increase their skills in using technology and making use of it in the reporting process. A study in Nigeria indicated that the inclusion of a financial reward for all reported acute flaccid paralysis cases greatly encouraged staff, surveillance coordinators, clinicians, and community informers to detect and report AFP; there was a marked increase in reports of AFP cases after the provision of the financial reward, [14].

Although the Internet has become necessary to speed up data reporting in many disease surveillance programs, we note Internet service is absent at all levels of reporting sites. This has become an urgent necessity to facilitate work procedures and perform activities. A Chinese study stated that the existence of an online platform facilitates the process of reporting cases, [8]. Given that all employees have mobile phones that can be provided with some assistance and some training courses on the use of technology in the investigation program in the River Nile State, we can help those in charge of the program in developing the system, upgrading it and keeping pace with events. Especially since there is good infrastructure, trained, cooperative and highly qualified human resources in performing their activities and monitoring work, and this is evidenced by their possession of very excellent information on the definition of AFP case, filling in the investigation form, collecting stool samples and the follow-up procedure. They also keep their files and report regularly to the higher authorities.

Through this study, it was that most of the system requirements are complete, such as having a strategy for the system and a plan for its evaluation, laws and regulations, coordination between localities and reporting sites, job description and training. This is in full agreement with what has been stated by the WHO as the structure of the surveillance system is defined by legislation (laws, and regulations, including IHR 2005), the strategy for implementing activities, the implementers and stakeholders, and how they relate to each other and the various networks and partnerships. The indicators that measure different aspects of the structure of a system constitute part of the evaluation indicators, [15].

The results of the activities’ performance showed that there is broad agreement with the general standards of the surveillance program, as there was an easy-to-use AFP field guide for all workers in surveillance activities at all levels, containing guidelines for writing the report and the decision-making process with the presence of focal persons in all reporting sites. In general, the resources available in the surveillance offices of localities were more than those available in health facilities, where not every health facility had a separate office for the surveillance activities; only 68% had a separate room for surveillance activities. There was access to public transport or electronic services that help to report cases, and although the localities have special vehicles for the surveillance system, the absence of support and continuous breakdowns affects the supervision activities [11].

The current study results illustrated that Calculators were available for data analysis in all locality’s offices and 36% of health facilities, all localities had computers, and only 10% of health facilities had a computer. 24% of health facilities have a phone for program activities. Nevertheless; the reporting process is ideal, using personal phones, which were available to all respondents. There are no printers in all health facilities and localities, just at the state level. There are also no internet lines at all levels; this confirms that the program still adopts the traditional methods in the reporting process, which is its use of the paper reporting system to collect data from reporting sites. In spite of that, the system was of high quality in terms of performance indicators, as the efficiency indicators were in conformity with the requirements of the WHO standards. Thus, the system is considered to have high quality in the performance of activities, especially with regard to indicators of completeness and sensitivity, where efficiency means the completion of the weekly and monthly reports by more than 80%. While sensitivity means reporting at least two cases per year for every100,000 people of the population under 15 years old, [16]. That is exactly what was found in the River Nile State, this sensitivity may increase if technology is widely introduced into the program with the increase in reporting sites and the development of the community surveillance system by giving volunteers modern work aids to encourage them to work efficiently.

Finally, if the surveillance activity finds support in addition to its available capabilities, it can be used to monitor a number of other diseases on a larger scale in addition to acute flaccid paralysis, especially those diseases targeted by vaccination, which may provide a database that easily guarantees knowledge and evaluation of the health situation in the community in the state Thus helping decision-makers in the intervention process in a timely manner, This is what was referred to by the reference, [8]; Where It Was Mentioned: Trained and experienced AFP surveillance officers can use AFP surveillance assets to help build integrated disease surveillance systems, providing opportunities to enhance comprehensive immunization program. This is particularly important for child immunization programs in low-income countries. On taking a closer look at selected countries, it becomes clear that polio origins not only help build capacity for non-polio-related programs, but provide the actual capacity for these efforts; In some cases, the origins of polio are the only resource-supporting functions in the country. For these reasons, in resource-poor countries, international partners and country governments must collaborate to support key monitoring functions. Without such a shift, polio eradication, control, and elimination of other vaccine-preventable diseases will suffer.

The existence of open borders between Sudan and a number of African countries in which polio viruses are still spreading necessitates that we have an integrated system for monitoring and support it with material and human resources at the level of all states in order to protect children from the transmission of the polio virus, despite Sudan was free of the virus for a decade ago. However, in 2020, during this study and using the effective state surveillance system, the Federal Ministry of Health notified in August, 2020 of the detection of circulating vaccine-derived poliovirus type 2 (cVDPV2) through the AFP surveillance system. According to the notification, the virus is genetically related to Chad. Two cases of acute flaccid paralysis were reported, Both children had taken the last dose of Oral Polio Vaccine (types 1 and 3) in 2019. Investigations indicated that these two cases were linked to the type 2 circulating vaccine-derived poliovirus (CVDPV) that appeared and was first detected in October 2019, which is currently circulating in Chad and Cameroon. 11 others suspected cVDPV2 cases were also confirmed, and field investigation reports were compiled; these cases are found in the following states: Red Sea, West Darfur, East Darfur, White Nile, River Nile and Gezira. Thus, between 9 and 26 August 2020, a total of 13 cVDPV2 cases were reported [17].

The ability of the surveillance system to detect those cases confirms its quality, but it needs to be strengthened in some aspects, especially cold chain and internet services, and the impact of that which appeared during the COVID-19 pandemic, may have an impact on those reported cases, although the services continue to be provided, while routine immunization activities in operating health centers have been suspended, the polio supplementary immunization activities throughout Sudan have been suspended at second half of 2020, as well as due to the difficulty of movement, lack of transportation and movement restrictions, stool samples collected from acute flaccid paralysis cases are preserved at the state level. The public health laboratory was provide by internet services by the World Health Organization to avoid delays in reporting and to ensure the timing of acute flaccid paralysis and environmental surveillance reports, which resulted in instant notifications of cases via email, but the investigation forms and stool samples were kept in cold chains at state level, This requires that all the elements that maintain the quality of the system in the state are available, have the ability to perform routine work and are prepared to respond to emergencies [18].

## Conclusion

Sudan continues to report cases of imported polio from other countries, demonstrating the need to strengthen the system, Although the surveillance system has the ability to detect cases, there is a problem with the open borders of the country. It does not seem that immunization alone is able to stop imported cases, which requires more integration of efforts among countries to stop the transmission of the polio virus.

## Data Availability

The datasets used and/or analyzed during the current study are available from the corresponding author on reasonable request.
